# Alteration of the Total Cellular Glycome during Late Differentiation of Chondrocytes

**DOI:** 10.3390/ijms20143546

**Published:** 2019-07-19

**Authors:** Kentaro Homan, Hisatoshi Hanamatsu, Jun-ichi Furukawa, Kazue Okada, Ikuko Yokota, Tomohiro Onodera, Norimasa Iwasaki

**Affiliations:** 1 Department of Orthopaedic Surgery, Faculty of Medicine and Graduate School of Medicine, Hokkaido University, Kita 15, Nishi 7, Kita-ku, Sapporo 060-8638, Japan; 2 Department of Advanced Clinical Glycobiology, Faculty of Medicine and Graduate School of Medicine, Hokkaido University, Kita 21, Nishi 11, Kita-ku, Sapporo 001-0021, Japan

**Keywords:** chondrocyte hypertrophy, glycomics, glycoconjugate, glycosphingolipid, glycosaminoglycan

## Abstract

In normal articular cartilage, chondrocytes do not readily proliferate or terminally differentiate, and exhibit a low level of metabolism. Hypertrophy-like changes of chondrocytes have been proposed to play a role in the pathogenesis of osteoarthritis by inducing protease-mediated cartilage degradation and calcification; however, the molecular mechanisms underlying these changes are unclear. Glycans are located on the outermost cell surface. Dynamic cellular differentiation can be monitored and quantitatively characterized by profiling the glycan structures of total cellular glycoproteins. This study aimed to clarify the alterations in glycans upon late differentiation of chondrocytes, during which hypertrophy-like changes occur. Primary mouse chondrocytes were differentiated using an insulin-induced chondro-osteogenic differentiation model. Comprehensive glycomics, including N-glycans, O-glycans, free oligosaccharides, glycosaminoglycan, and glycosphingolipid, were analyzed for the chondrocytes after 0-, 10- and 20-days cultivation. The comparison and clustering of the alteration of glycans upon hypertrophy-like changes of primary chondrocytes were performed. Comprehensive glycomic analyses provided complementary alterations in the levels of various glycans derived from glycoconjugates during hypertrophic differentiation. In addition, expression of genes related to glycan biosynthesis and metabolic processes was significantly correlated with glycan alterations. Our results indicate that total cellular glycan alterations are closely associated with chondrocyte hypertrophy and help to describe the glycophenotype by chondrocytes and their hypertrophic differentiation. our results will assist the identification of diagnostic and differentiation biomarkers in the future.

## 1. Introduction

Maturation of chondrocyte via different phases results in hypertrophy [1,2]. Although chondrocyte hypertrophy induces matrix calcification, which is essential for bony elongation and repair of the skeleton, ectopic hypertrophy of articular chondrocytes has been implicated in the pathogenesis of osteoarthritis (OA) [3,4,5]. Remarkably, some characteristics of OA, namely articular chondrocyte proliferation, an increase in expression of hypertrophy markers (MMP13 and type X collagen), matrix remodeling by proteases, vascularization, and focal calcification, resemble those of chondrocyte differentiation during skeletal development by endochondral ossification [5]. Therefore, a better understanding of the processes underlying chondrocyte hypertrophy in articular cartilage may improve the management of OA.

Understanding of the molecular and cellular mechanisms that control chondrocyte hypertrophy has rapidly increased in recent years. Functional genomics and proteomics during cellular differentiation have also been intensively studied [6,7,8,9,10,11,12]. However, despite this detailed knowledge of hypertrophic differentiation, the molecular mechanisms that mediate the initiation and progression of articular cartilage degeneration and ultimately lead to OA remain poorly understood. A variety of intricately arranged glycoconjugates on the surfaces of mammalian cells, such as glycoproteins, glycolipids, and proteoglycans, seem to change in close association with each other during the differentiation process. The composition of the oligosaccharide chains on the cell surface is known to begin to change prior to alteration of cellular morphology, not only during the differentiation process, but in pathogenesis of some organs [13,14]. We previously reported that the expression levels of high-mannose (HM)-type N-glycans are up-regulated in mouse chondroprogenitor cells at the late stage of differentiation [15]. The levels of these glycans are significantly decreased in human OA cartilage [16]. On the other hand, glycosphingolipids (GSLs) in cartilage help to maintain chondrocyte homeostasis and differentiation. Gangliosides are the most abundant GSLs, and we previously revealed that loss of gangliosides suppresses in vitro differentiation of murine primary chondrocytes into the terminal hypertrophic state [17]. Previous studies reported that the amount of gangliosides decreased in human OA [18,19], and some of the GSLs play suppressive roles in OA pathogenesis [17,20]. These results suggest that alteration of glycans is associated with the pathogenesis of OA, especially in relation to the hypertrophic process. Given that protein glycosylation is a common post-translational modification and most cellular proteins are modified by N-glycans, O-glycans, and glycosaminoglycans (GAGs) [21], a systematic overview of all the major classes of glycans in cellular glycoconjugates would help to further characterize this differentiated phenotype.

To comprehensively understand the alteration of glycans during chondrocyte differentiation, we developed a standardized glycomics protocol based on a simple chemical enrichment method, namely, a glycoblotting method that allows rapid and large-scale [22,23,24] enrichment analysis of cellular glycans. Furthermore, β-elimination in the presence of pyrazolone analogues (BEP) was developed for O-glycome analysis [25,26]. This study provides information about the glycome that can be used to visualize the entire complement of sugars, including N-glycans, O-glycans, GAGs, GSL-associated glycans, and free oligosaccharides (fOSs), in hypertrophic chondrocytes. Our results reveal complementary alterations in N-glycans and fOSs as well as striking changes in the quantities and qualities of O-glycans and GAGs.

## 2. Results

### 2.1. Chondrocyte Hypertrophy Culture Model

**Figure 1:**
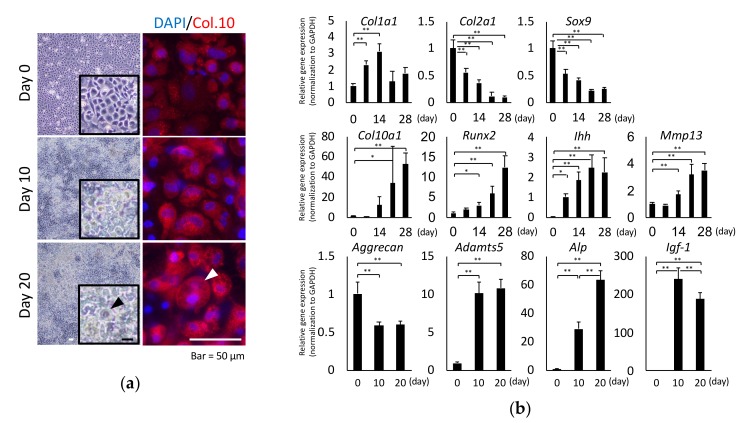
Morphological and gene expression changes during hypertrophic differentiation. (**a**) Microscopic observation of hypertrophic-like changes in chondrocytes (allows). Note that the volume of the cell is drastically increased. Chondrocytes were co-stained with 4′ 6-diamidino-2-phenylindole (DAPI) and type X collagen immunoglobulin G (IgG). Scale bar = 50 μm. (**b**) Time course changes in the expression of marker genes during the hypertrophic differentiation of chondrocytes. The increased expression of hypertrophic markers was significant after 14 days (*Runx2*: *p* < 0.01; *Mmp13*: *p* < 0.0001; *Ihh*: *p* < 0.001) and 21 days (*Col10a1*: *p* < 0.0001) compared with expression before induction (0 day). Gene expression levels were measured by quantitative real-time polymerase chain reaction (PCR) (*n* = 3 per group; * *p* < 0.05; ** *p* < 0.01).

Murine primary chondrocytes were used for a well-established model [17,27] of in vitro differentiation. These cells displayed enhanced hypertrophic differentiation, as evidenced by the increased number of type X collagen-stained nodules after 10 days of differentiation (Figure 1a). Characteristics of hypertrophic chondrocytes, such as morphological changes from polygonal to round or an increase of intracellular volume, occurred from day 10 onwards. These cells also expressed type X collagen, which is frequently used as a marker of hypertrophic differentiation. The increased gene expression of *Col10a1* by quantitative real-time reverse transcription-polymerase chain reaction (qPCR) appeared on day 14, and the expression of other differentiation markers (*Mmp13*, *Runx2*, *Indian hedgehog* (*Ihh*)) and cartilage anabolic factors (*Sox9*, *Col2a1*) also showed characteristic fluctuations between day 7 and day 14 (Figure 1b). It was reported that an elevation of alkaline phosphatase (ALP) activity and the mRNA expression of type X collagen induced during osteogenic differentiation of ATDC5 cells were first observed from day 16 to day 20 after the induction of insulin [28]. Therefore, we focused on three time points, namely, days 0, 10, and 20, to comprehensively analyze the total cellular glycome during hypertrophic differentiation. These time points were consistent with a marked increase in the *insulin-like growth factor 1* (*Igf-1*), a promoter of growth and matrix synthesis by chondrocytes, which reflects increases in the levels of representative aggrecanase (*Adamts5*) and mineralization markers (*Alp*) (Figure 1b).

### 2.2. Total Glycomic Analysis of Chondrocytes During Hypertrophy

**Figure 2:**
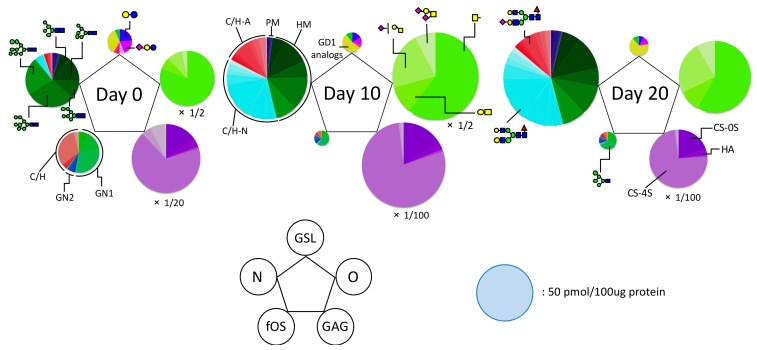
Comprehensive glycomic profiles during hypertrophic differentiation. Pentagonal cellular glycomic notations represent the entire glycome of each time point of hypertrophic differentiation and indicate the relative abundance and diversity of each class of glycoconjugate. Pie charts at the vertices of the pentagon correspond to the glycan expression profiles of N-glycans, free oligosaccharides (fOSs), glycosaminoglycans (GAGs), glycosphingolipid (GSL)-glycans, and O-glycans. The size of each circle and its constituent colors reflect the absolute glycan quantity (50 pmol/100 μg of protein) and the glycan substructures, respectively. The sizes of the circles representing the O-glycans and GAGs contents increased by 2-fold and 10-fold (area ratio), respectively. fOS is constituted of neutral oligosaccharides possessing one GlcNAc (GN1) or two GIcNAc (GN2) at the reducing endGN, C/H: complex/hybrid-type fOSs, N-glycans can be structurally classified into pauci-mannose (PM), high-mannose (HM), and complex/hybrid (C/H)-types. Two major components of GAGs are chondroitin sulfate (CS) and hyaluronic acid (HA). The estimated glycan structures are presented as follows: green circle, Man; yellow circle, Gal; blue square, GlcNAc; yellow square, GalNAc; red triangle, Fuc; purple diamond, Neu5Ac; and open diamond, Neu5Gc. The estimated glycan structures are shown in Table S1.

The purpose of this study was to define the total glycome of chondrocytes during hypertrophy. For total cellular glycomic analysis, we retrieved all of the previously reported elementary cellular glycomes [23], and then obtained glycomic data on chondrocytes and chondrocytes undergoing hypertrophic differentiation. As shown in Figure 2, the total glycomic profile of each cell state was illustrated based on the absolute amount of each type of glycan and glycan substructure. Pie charts at the vertices of each pentagon correspond to the glycan expression profiles of N-glycans, fOSs, GAGs, GSL-glycans, and O-glycans. In this study, we observed significant alterations in all classes of glycoconjugates (i.e., GSLs, N- and O-glycans, chondroitin sulfate (CS), heparan sulphate (HS), and fOSs) in chondrocytes during hypertrophy. GAGs and O-glycans were abundant in chondrocytes, accounting for more than 94.8% of the glycoconjugates in the total cellular glycome (Table S1). Compared with day 0, however, on day 10 there was an increase in N- and O-glycans and GAG disaccharides, but a decrease in fOSs and GSL.

### 2.3. Cluster Analysis

**Figure 3:**
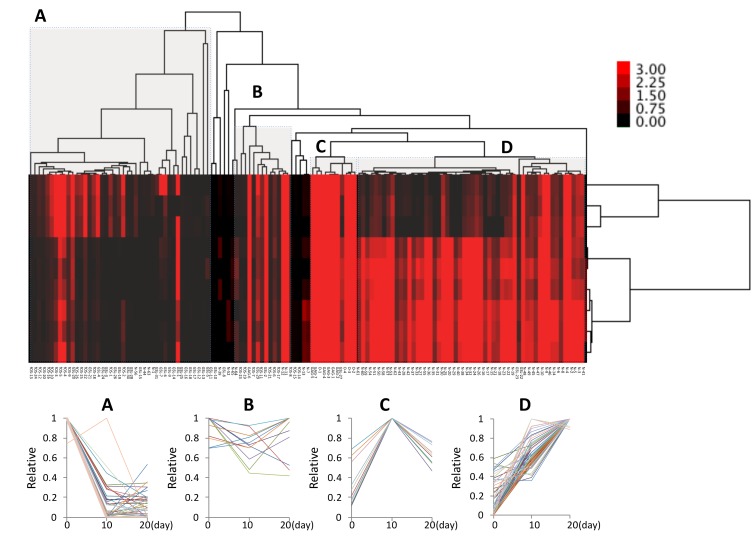
Classification of cells and glycans based on unsupervised cluster analysis. The absolute amount of each glycan (pmol/100 μg of protein) was analyzed using Cluster 3.0 Software. The relative abundance and the amount of each glycan (classified into Group A, B, C, or D) is shown in Table S1 and Figure S3.

Hierarchical clustering analysis based on quantitative glycomic profiles of N-, O-, and GSL-glycans, fOSs, and GAG disaccharides resulted in the classification of model cells (Figure 3). The total glycan expression (profile) of day 0 cells was different from the cluster of day 10 and day 20 cells. These results indicated that glycan biosynthesis and metabolic processes were different in chondrocytes during hypertrophy, although there were also similarities between day 10 and day 20. Hypertrophic differentiation could be broadly classified into four categories based on the relative increase in the expression of glycans, as follows: the expression of glycans in Group A, which included fOS and GSL-glycans, tended to decrease with differentiation; Group B was composed of stably expressed glycan in the process of hypertrophy and included all glycan types other than O-glycans; O-glycans and GAG were clustered in Group C, indicating a close similarity among the different stages of differentiation-related glycophenotypes; and Group D included many clades, most of which were occupied by N-glycans, showing an increasing trend with differentiation. Cluster analysis showed that the levels of various N-glycans progressively increased with hypertrophic progression, whereas those of GSL-glycans and fOSs significantly decreased. Changes in the levels of O-glycans and GAG were transient. The cluster classification of the expression of different glycans is shown in Table S1.

### 2.4. N-Glycans during Hypertrophic Differentiation

**Figure 4:**
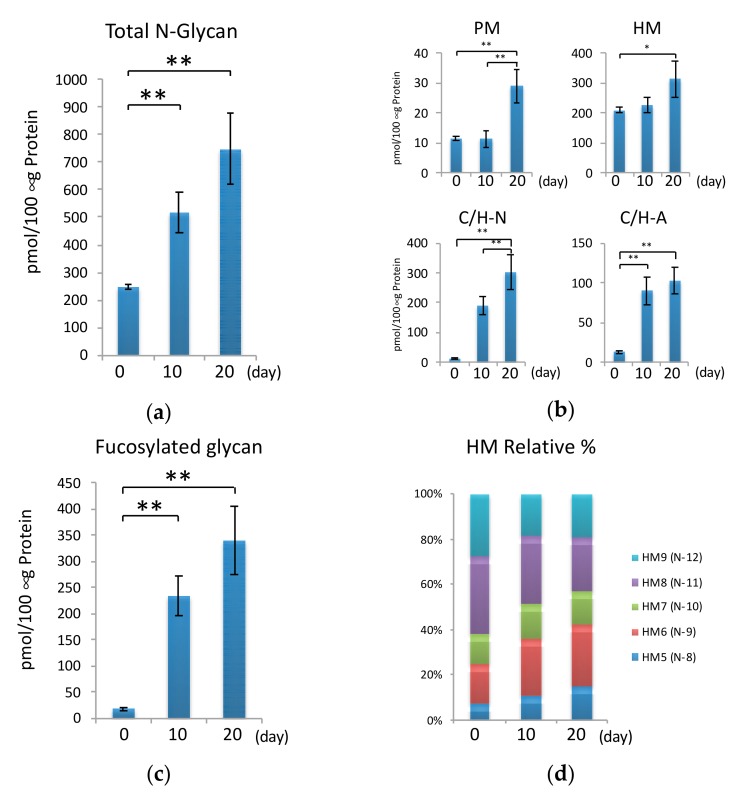
Total cellular N-glycome during hypertrophic differentiation. (**a**) Total amount of N-glycan in cells at each time point (day 0, 10, and 20). (**b**) Amount of each class of glycan. Pauci mannose (PM); high mannose (HM); complex/hybrid neutral (C/H-N); and complex/hybrid acidic (C/H-A)-types. (**c**) Total amount of fucosylated N-glycan in C/H-N- and C/H-A-types. (**d**) Relative amount of high mannose-type N-glycans. At each time point, the ratio of the high-mannose glycan (HM5-9) was calculated when the total value of the HM glycan was taken as 100%. The results are expressed as the means ± standard deviation (S.D.). * 0.01 < *p* < 0.05; ** *p* < 0.01. All MALDI-TOF-MS measurements were independently carried out (*n* = 3). The prepared glycan structures are listed in Supplementary Table S1.

**Figure 5:**
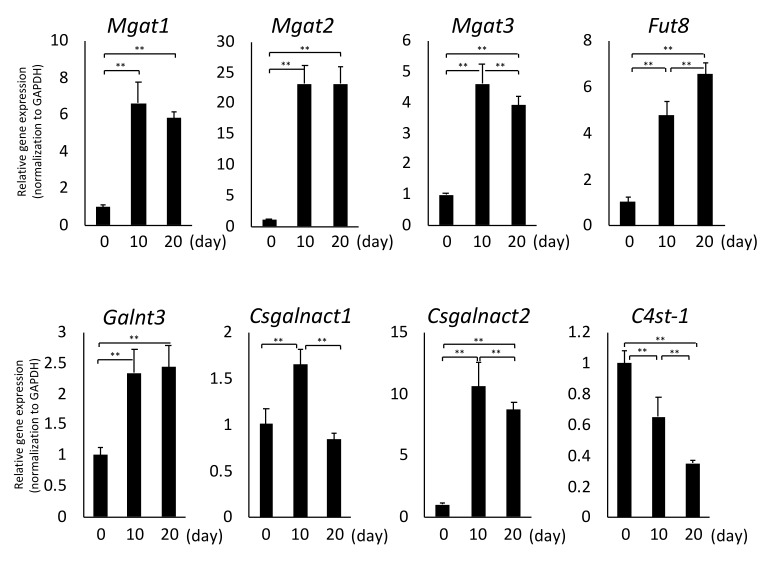
Gene expression levels of glycosyltransferases during hypertrophic differentiation involved in glycan biosynthesis and processing. The mRNA expression levels on the indicated days as determined by quantitative RT-PCR. Data represent the relative gene expression in chondrocytes on each day compared with that in cells on day 0 (set to 1.0 separately for each target gene; *n* = 3 per group; * 0.01 < *p* < 0.05; ** *p* < 0.01). The results are expressed as the means ± S.D.

The total N-glycan amount increased significantly during hypertrophic differentiation, as shown in Figure 4a. N-glycans can be classified into pauci-mannose (PM; Man1-4GlcNAc2 Fuc0-1), HM (Man5-9 GlcNAc2), neutral complex/hybrid (C/H-N), and sialylated complex/hybrid (C/H-A)-types according to their structure. Although the total amount of PM- and HM-type N-glycans was not affected between day 0 and day 10, these glycan types increased significantly on day 20. This tendency was not observed for the other N-glycan types. The expression levels of C/H-N- and C/H-A-type glycans increased significantly after hypertrophic stimulation (Figure 4b). Moreover, the C/H-N-type glycan containing fucose drastically increased after day 10 (Figure 4c). By matrix-assisted laser desorption/ionization (MALDI) time-of-flight/time-of-flight (TOF/TOF) analysis of highly expressed C/H-N-type glycans (N-30, -32, and -34), we found most C/H-type glycans to contain core fucose as shown in Figure S1. These results raise the possibility that glycan processing and fucosylation occurred during chondrocyte hypertrophy. Therefore, we next investigated the gene expression level of *Mgat1-3*, which is associated with N-glycan processing, and the core fucose transferase *Fut8*. Consistent with the results of mass spectrometry analysis, the expression of *Mgat1-3* and *Fut8* increased significantly (Figure 5). With regard to the ratios of the HM-type glycans, the relative amounts of HM5 (N-8) and HM6 (N-9) increased, whereas those of HM8 (N-11) and HM9 (N-12) decreased, during hypertrophic differentiation (Figure 4d). These results are also in good agreement with the gene expression profile shown in Figure 5.

### 2.5. fOSs during Hypertrophic Differentiation

**Figure 6:**
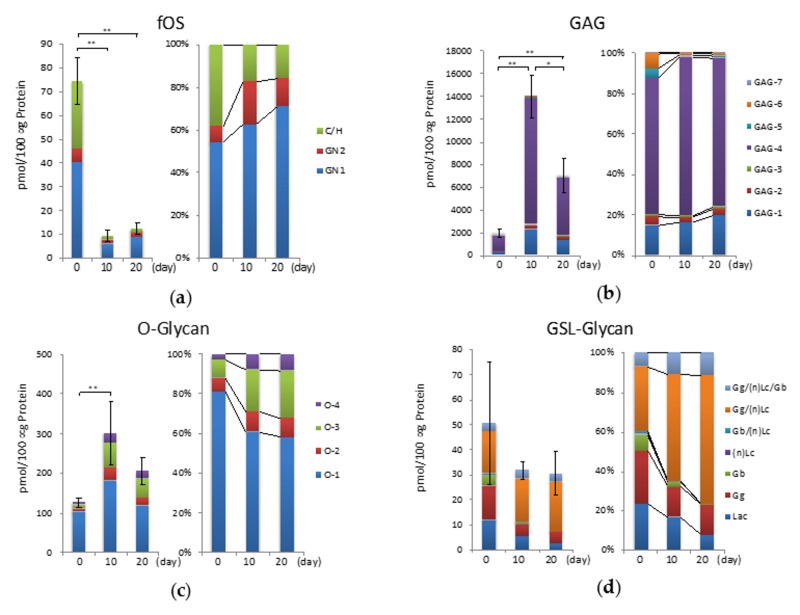
Total cellular glycomes of fOS, GAG, and O- and GSL-glycan during hypertrophic differentiation. (**a**) The absolute and relative amounts of fOS types. (GN1, fOS with a single GlcNAc residue; GN2, N,N-diacetylchitobiose moiety of fOS; C/H, Complex/hybrid-type fOS.) (**b**) The absolute and relative amounts of disaccharidic GAGs. Analysis of GAGs was performed with HPLC. (**c**) The absolute and relative amounts of O-glycans. (**d**) The absolute and relative amount of the GSL-glycan series. (Lac, Laccer; Gg, Ganglioside series; Gb, Globo series; (n)Lc, (neo)Lacto series). The results are expressed as the means ± S.D. * 0.01 < *p* < 0.05; ** *p* < 0.01. All MALDI-TOF-MS and HPLC measurements were independently carried out (*n* = 3). The prepared glycan structures are listed in Supplementary Table S1.

HM-type fOSs are generated from either misfolded proteins or dolichol-linked oligosaccharides. These fOSs can be classified into two structural types, namely, the Gn2-type (with chitobiose at the reducing end) and the Gn1-type (with a single GlcNAc at the reducing end). Recently, we observed complex-hybrid (C/H)-type fOSs in the serum and various cells [24,29]. Therefore, fOSs were analyzed in chondrocytes, as well as in chondrocytes undergoing hypertrophic differentiation, and then classified as the Gn1-type, Gn2-type, or C/H -type fOSs type. In this study, we found that free glycans derived from N-glycans accumulated in chondrocytes. The total amount of fOSs decreased significantly by one-eighth after hypertrophic differentiation (Figure 6a). Specifically, C/H-type fOSs were abundant in murine primary chondrocytes, which was largely due to an increase in fOS-22 (HexNAc_2_ Neu5Ac_1_ + Man_3_GlcNAc_1_). During hypertrophic differentiation, however, the relative amount of C/H-type fOS at day 10 and day 20 appeared to decrease by more than 50% compared to day 0 (graph on the right) (Figure 6a). Furthermore, the amount of fOSs decreased significantly, although that of N-glycans increased after hypertrophic differentiation.

### 2.6. GAGs during Hypertrophic Differentiation

In murine primary chondrocytes, GAGs were the major component in cellular glycoconjugates, and the total amount was 1918 pmol/100 μg of protein (Figure 6b). On day 10 of hypertrophic differentiation, the expression of total GAGs was at its highest. In murine chondrocytes, GAG-4, the monosulfated disaccharide at the 4-position of GalNAc, was the major component of CS (approximately 70% of the total amount of GAG), which is in good agreement with a previous study [30]. HS- and hyaluronate (HA)-type GAGs were minor components in chondrocytes and chondrocytes undergoing hypertrophic differentiation. Changes in the percentage of GAG contents due to hypertrophic differentiation were moderate, regardless of the decreased expression of sulfotransferase (*C4st-1*) and increased expression of *Csgalnact* (Figure 5).

### 2.7. O-Glycans During Hypertrophic Differentiation

O-glycans were released from cellular glycoproteins and simultaneously labeled by microwave-assisted β-elimination in the presence of pyrazolone analogues (MW-assisted BEP) as previously reported [26]. The expression patterns of O-glycans were similar to those of GAGs. Four mucin-type O-glycans were detected in this study. The most abundant glycan was Tn (O-1, HexNAc_1_), the main component of cartilage in chondrocytes [31]. Sialyl-T (O-3, Hex_1_HexNAc_1_Neu5Ac_1_) and di-sialyl-T (O-4, Hex_1_HexNAc_1_Neu5Ac_2_) antigens increased nearly 6-fold on day 10 of hypertrophic differentiation (Figure 6c). The ratio of acidic glycans increased 3-fold on day 20 (Figure 6c). Real-time RT-PCR analysis confirmed the high expression of *Galnt3* associated with the O-glycosylation of serine and threonine residues for mucin (Figure 5). Other O-glycan types, such as O-mannose and O-fucose, could not be detected in this study.

### 2.8. GSL-Glycans during Hypertrophic Differentiation

GSLs are ubiquitous components distributed on the cell membranes of organisms. We quantified 34 GSL-glycans in murine chondrocytes. The total amounts of GSL-glycans and fOSs decreased during hypertrophic differentiation (Figure 6d). The Gg/(n)Lc series increased significantly with hypertrophy, which was due to an increase in Hex_3_HexNAc_1_Neu5Ac_2_ (GSL-22) (Figure S2). For example, GSL-22 is a Gg series-glycan, which has many structural isomers that are presumed to be GD1 analogues. GM3 (GSL-2) and GD1 analogues were the most abundant gangliosides in chondrocyte isolated from immature mice, which was consistent with the previous report of GSL composition in mouse cartilage [17] (Figure S2). On the other hand, the Gg series decreased on day 10 of hypertrophic differentiation. The amount of GM3, a precursor of GD1 analogues and the main component of murine chondrocytes, decreased.

## 3. Discussion

There are several models to evaluate differentiation of chondrocytes into a hypertrophic state. In the chondrocyte micromass model, which was originally used as a growth plate model for cartilage differentiation [32,33,34], chondrogenic differentiation is enhanced due to considerable cell-cell interactions. However, this model tends to induce dedifferentiation of chondrocytes, especially when cells are cultured for more than 3 weeks. Chondrogenic growth factors, including insulin, bone morphogenetic protein 2 (BMP-2), and transforming growth factor β1 (TGF-β1), enhance chondrocyte hypertrophy as well as chondrogenic differentiation [35,36,37]. A previous study, which compared the use of BMP-2 and insulin-transferrin-selenium (ITS) to generate a chondrogenic murine micromass cell culture system, reported that BMP-2 treatment induces a more chondrogenic phenotype and ITS treatment favors maturation and hypertrophy of chondrocytes [38]. In addition, a combination of insulin, transferrin, and selenium prevents chondrocyte dedifferentiation and promotes differentiation in monolayer culture [39]. In the current study, primary chondrocytes had to be isolated and cultured such that they retained their cell type and differentiated into a hypertrophic state; therefore, we decided to culture mouse primary chondrocytes under differentiation conditions in the presence of insulin. The phenotype of chondrocytes was characterized by analyzing specific hypertrophy markers and observing cellular morphology. Chondrocyte hypertrophy was observed after cells became confluent via a cellular condensation process, resulting in formation of cartilage nodule-like cell aggregates. This occurred concomitant with type X collagen gene expression and a dramatic elevation in ALP activity. *Sox9* and *Runx2* gene products are critical master transcriptional regulators of chondrocyte differentiation and regulate chondrogenesis and osteogenesis. Analysis of these two genes demonstrated that the main steps of chondro-osteogenesis in vivo were replicated at the cellular level in this model. The decrease of the expression levels of *Col2a1* and *Sox9* is reminiscent of a tendency towards dedifferentiation; whereas these alterations are also consistent with the characteristics of hypertrophy [40,41]. The expression level of *Col1a1* temporarily increased and subsequently returned to the same expression level on day 0, suggesting that hypertrophic differentiation occurred rather than dedifferentiation in this culture system. Other studies have used the same method to investigate chondrocyte hypertrophy [27,42]. Insulin-induced chondrocyte differentiation provided an excellent model to study the molecular mechanism underlying regulation of articular chondrocyte differentiation during hypertrophy over time.

The total glycome was recently reported to reflect highly cell-specific profiles [23,24]. The current study is the first to analyze the cellular glycome including all major classes of glycoconjugates upon late differentiation of chondrocytes, during which hypertrophy-like changes occur. Cluster analysis identified four distinctive expression patterns for each glycan type, and N-linked glycan chains had the most characteristic expression pattern (Group D). Expression of HM-type N-glycans was up-regulated at a late stage of murine chondroprogenitor cell differentiation. The amount of HM5 (N-8) increased five-fold during chondrogenic differentiation and remained at this elevated level in mature chondrocytes [15]. Yan et al. revealed that chondrocytes exposed to concanavalin A, which binds specifically to HM-type structures, switch from the resting to the hypertrophic stage [43,44]. Our results showed that the amount of HM5 increased when hypertrophic chondrocytes underwent differentiation. The level of a C/H-type and fucosylated glycan chain located downstream of the N-Glycan biosynthesis increased, indicating that secretion and transport to membranes are activated along with hypertrophy in chondrocytes. *Man2a1* and *Mgat2* convert oligomannosides to the complex type. The glyco-phenotype of osteoarthritic cartilage and human chondrocytes involves the synthesis of a complex-type N-glycan (Gnt-II by the *Mgat2* gene) with core substitutions (FUT8, Gnt-III by the *Mgat3* gene) [45]. Similarly, gene expression of *Mgat2* and *Fut8* increased during hypertrophic differentiation of chondrocytes (Figure 5). Our results suggest that primary chondrocytes at day 0 constantly synthesize and degrade N-glycans, which are utilized to synthesize complex-type N-glycans as hypertrophic differentiation progresses. In addition, the quantity of fOSs decreased with the structural changes of N-glycans. Furthermore, the ratio of the quantity of fOSs to N-glycans dramatically decreased by 3.5% from 57.8% upon hypertrophic differentiation. fOSs are generated as a result of N-glycoprotein catabolism; during protein N-glycosylation in mammalian cells, fOSs are generated from dolichol-linked oligosaccharides (DLOs) and misfolded glycoproteins. Although HM-type fOS (GN1, GN2) is known to be produced in endoplasmic reticulum-associated degradation (ERAD), we also detected unique C/H-type fOSs (Table S1) with poorly understood biosynthetic pathways. The rapid decrease in expression of this C/H-type fOS was a prominent feature of hypertrophy.

According to cluster analysis, O-glycans and GAGs were classified into a single clade in the tree diagram and their expression levels increased at day 10 and decreased at day 20 of hypertrophic differentiation. These entities correspond to mucin and aggrecan, which are major extracellular matrix proteins in articular cartilage. Expression of O-glycans and GAGs was synergistically altered together with that of *Igf-1*, which is potent factor in matrix synthesis. Regarding glycosyltransferases, we evaluated the mRNA expression pattern of *CS glycosyltransferases (Csgalnact1, 2)*, which are responsible for the initiation and prolongation of chondroitin sulfate synthesis [30,46]. Our results revealed that the expression pattern of *CS glycosyltransferases* was synchronized with those of O-glycans and GAGs, suggesting that alterations in the levels of O-glycans and GAGs reflect changes in matrix production by chondrocytes during hypertrophic differentiation.

Cluster analysis also revealed that the total number of GSLs decreased during hypertrophic differentiation. Previous studies reported that the ganglioside content of OA cartilage is decreased in human [18,19]. The alteration of GSLs during hypertrophy, which is a characteristic feature of OA, is consistent with previous findings in human OA. In addition, the amount of GM3 decreased, while that of GD1 analogues increased. We previously reported that GM3 plays suppressive roles in OA [17,20,47,48,49]. GD1 analogues are downstream of GM3 in the biosynthetic pathway; therefore, the increase in GD1 analogues during hypertrophy observed in the current study was likely due to structural alteration of GM3. Based on the previous and present results, structural alteration of GM3 may play an important role in the pathogenesis of OA. The methods we have employed extract glycoproteins of the whole cell, including membrane, cytoplasm, nucleus, and so on. We speculate that the alterations of glycosphingolipids primarily reflected those in cell membranes since glycosphingolipids mainly exist in the outer layer of cell membranes [50]. Other glycoconjugates exist in membrane and cytoplasm, and hence we cannot distinguish the distribution of them in this study.

Expression of glycoconjugates dramatically changed upon chondrocyte hypertrophy. Our comprehensive profiling did not include analyses of keratan sulfates due to technical difficulties and cerebrosides because Rhodococcus endoglycoceramidase (EGCase)s were used. In addition, alteration of cell size or normalization of the amount of glycoconjugates by cell size was not performed in this study. It is necessary to take this point into consideration with regard to the alterations in the amount of glycoconjugates with differentiation. Despite this limitation, our study provides new insights into alterations of glycoconjugates during hypertrophic changes. Although the biological consequences of these alterations remain to be elucidated, our results will assist the identification of diagnostic and differentiation biomarkers, as well as the screening of therapeutic targets in the future.

## 4. Materials and Methods

### 4.1. Experimental Animals and Materials

Wild-type C57BL/6 (5-day-old) mice were purchased from Japan SLC, Inc. (Hamamatsu, Japan). All experiments were performed according to a protocol approved by the Institutional Animal Care and Use Committee of the Hokkaido University Graduate School of Medicine (Sapporo, Japan) (approved number: 17-0059, 10 May 2018). EGCase I was prepared as previously described [51]. BlotGlyco R beads and aminooxy-WR (aoWR) reagent were obtained from Sumitomo Bakelite Co., Ltd. (Tokyo, Japan). Disialyloctasaccharide (A2GN1), 1-phenyl-3-methyl-5-pyrazolone (PMP), and N, N, N, N-tetraacetyl chitotetraose (GN4) were purchased from Tokyo Chemical Industry (Tokyo, Japan). Other solvents and reagents were of the highest grade commercially available.

### 4.2. In Vitro Differentiation

Insulin-induced chondro-osteogenic differentiation of primary cells was performed to monitor differentiation of chondrocytes into a hypertrophic state in vitro over time [27,42,52]. This method involved isolating and culturing primary chondrocytes such that they retained their cell type and differentiated into a hypertrophic state [42]. Immature mouse chondrocytes were obtained from the knee joints of newborn mice as previously described [53]. Briefly, articular cartilage of the femoral condyles and tibial plateaus was isolated from mice and digested twice with 3 mg/mL collagenase D (Roche Applied Science, Mannheim, Germany) for 45 min, followed by 0.5 mg/mL collagenase D overnight. This protocol obviated the need for cell trypsinization, which leads to dedifferentiation, allowing cellular glycobiology investigations of fully differentiated primary chondrocytes. Chondrocytes were filtered through a sterile 48 μm cell strainer and cultured in DMEM/Ham’s F-12 (1:1) (Wako Pure Chem., Osaka, Japan) containing 5% fetal bovine serum (FBS; Nichirei, Tokyo, Japan), 100 units/mL penicillin G, and 100 g/mL streptomycin. Cells were cultured until subconfluent, which took approximately 2 days, and then induced to differentiate by adding 1× ITS universal culture supplement (containing insulin, transferrin, and selenous acid; Sigma-Aldrich, Tokyo, Japan) and 50 μg/mL ascorbic acid (Wako) in the presence of 2% FBS. Thereafter, cells were maintained in culture for the indicated durations, during which time the medium was changed every 48 h. Hypertrophic differentiation of chondrocytes was assessed by qPCR analysis and immunohistochemical staining for type X collagen. After washing the culture dish with cold phosphate-buffered saline (PBS), cells were scraped into cold PBS containing 10 mM EDTA and collected by centrifugation at 1000 ×*g* for 3 min. The pellet was washed five times with PBS to remove FBS and thereby minimize contamination of FBS-derived glycoproteins.

### 4.3. qRT-PCR Analysis

Total RNA was isolated from primary chondrocyte monolayer cultures using a standard protocol. Briefly, total RNA was isolated using TRIzol reagent (Life Technologies, Carlsbad, CA, USA), and after DNase treatment (Qiagen, Hilden, Germany) and column cleanup (Qiagen), 100 ng of total RNA was reverse-transcribed using the QuantiTect Reverse Transcription Kit (Qiagen), according to the manufacturer’s instructions. Gene amplification was carried out using the SYBR Green I-based RT-PCR Master Mix and the Thermal Cycler Dice TaKaRa Real-Time System II (model TP900; TaKaRa, Shiga, Japan) with gene-specific primers (Table S2). The relative messenger RNA (mRNA) expression level of each target gene was expressed as the Ct value of each gene normalized to the Ct value of the GAPDH gene using the ΔΔ*C*t method and the individual efficiency-corrected calculation method [54]. Data were normalized to the average mRNA level on day 0 (set at 1) and presented as means ± SEM.

### 4.4. Immunostaining for Type X Collagen

The distribution of type X collagen associating with chondrocyte differentiation was visualized with Alexa Fluor^®^ 555 goat-conjugated anti-rabbit IgG (1:200; Invitrogen, Carlsbad, CA, USA) after its validation (Supplementary Materials Figure S4). The negative control was incubated with normal rabbit serum (1:10,000; cat. no. S-5000; Vector Laboratories, Burlingame, CA, USA). Nuclei were stained with ProLong^®^ Gold Antifade reagent containing DAPI (Invitrogen).

### 4.5. Extraction of Glycoproteins, GSLs, and fOSs

Approximately 1.0 × 10^7^ cells were suspended in 200 μL of PBS and homogenized using a beads crusher (TAITEC, Saitama, Japan). To extract glycoproteins, a 4-fold volume of ethanol was added, and the sample was incubated at −30 °C for 16 h. The cell pellet and supernatant fractions were separated by centrifugation. The precipitates (containing 50 μg of protein) were dried and dissolved with water. Deglycosylation was performed as previously described [23]. The supernatants, containing GSLs and fOSs, were dried with a centrifugal evaporator. The resulting sheets were resuspended in 45 μL of 50 mM acetate buffer, pH 5.5, containing 0.2% Triton X-100 (Sigma-Aldrich). GSL-glycans were isolated by a 16 h enzymatic digestion using EGCase I at 37 °C, whereas fOSs were recovered from an EGCase I-free fraction.

### 4.6. Extraction of GAGs

A total of 20 μL of serum was delipidated and digested to GAG disaccharides. Detailed procedures and materials are provided elsewhere [55,56].

### 4.7. Extraction of O-Glycans

Extracted glycoproteins were subjected to BEP for O-glycan analysis. Detailed procedures and materials are provided elsewhere [25,26].

### 4.8. Glycoblotting

N-glycans, fOSs, GAGs, and GSL-glycans were subjected to glycoblotting. Detailed procedures and materials are provided elsewhere [22,26].

### 4.9. Quantitative Analysis by MALDI-TOF-MS

Purified N-glycans, fOSs, O-glycans, and GSL-glycan solutions were mixed with 2,5-dihydrobenzoic acid solution and subjected to MALDI-TOF-MS analysis, as previously described [23,26,57]. All peaks were selected using the SNAP algorithm within FlexAnalysis 3.0 Software, which fit the isotopic patterns to the matching experimental data. For high mass measurement accuracy (MMA), an external calibration was performed using N-glycans derived from human serum. The glycan compositions were manually determined by conducting database searches (i.e., a compositional search of the UniCarbKB database (http://www.unicarbkb.org/query) for fOSs and N- and O-glycans, and of the SphinGOMAP database (http://www.sphingomap.org/) for GSL-glycans). All previously deposited GSL-glycans in the SphinGOMAP database were extracted and compiled as an in-house database to allow searching by the *m*/*z* value and/or composition. The absolute quantification was obtained by comparative analyses between the MS signal areas derived from each glycan and the internal standard. For glycoblotting, glycans were subjected to on-bead methyl esterification to obtain sialylated oligosaccharides that were the chemical equivalents of neutral oligosaccharides [58]. As previously reported by us [57], the signal strength of a mixture of equal quantities of 14 aoWR-labeled glycans with molecular weights ranging from 772 to 2826 was similar, indicating that the structure and molecular weight had no significant effects on the signal strength. As some low-molecular-weight glycans (e.g., Hex2 of GSL-glycans, Hex1HexNAc1 of fOSs) were partially lost during the solid-phase extraction process, the absolute quantity was estimated by applying correction factors [57]. Fairly constant peak areas were also observed for the bis-PMP-labeled glycans, regardless of the differences in structure and molecular weight [26].

### 4.10. HPLC Analysis

2AB-labeled GAG disaccharides were analyzed using HPLC. Detailed procedures and materials are provided elsewhere [23,56].

### 4.11. Statistical Analysis

Data are expressed as means ± standard deviation (S.D.). The Welch *t*-test/Welch ANOVA, followed by the Tukey−Kramer multiple comparison test, was performed to determine significant differences between groups. *p*-values of less than 0.05 were considered significant. Data analysis was carried out using JMP^®^ Pro 13.1.0 Statistical Software (SAS Institute, Inc., Cary, NC, USA). Cluster analyses were performed with software Cluster 3.0 with a hierarchical clustering algorithm [59]. The calculated dendrogram and correlation matrix were visualized by using TreeView 1.1.6r4 software.

## 5. Conclusions

Integrated information obtained by comprehensive analysis of the glycoconjugate structure during chondrocyte hypertrophy indicates that total cellular glycan alterations are closely associated with chondrocyte hypertrophy. Obtained results in this study will assist the identification of diagnostic and differentiation biomarkers.

## Supplementary Materials

Supplementary materials can be found at https://www.mdpi.com/1422-0067/20/14/3546/s1.
