# Supplementary material for: Alteration of the Total Cellular Glycome during Late Differentiation of Chondrocytes
Source: Int J Mol Sci. 2019 Jul 19;20(14):3546. doi: 10.3390/ijms20143546 (PMC6678350; doi:10.3390/ijms20143546)
Supplement: Supplementary file 1 [file ijms-20-03546-s001.zip › ijms-532605-supplementary-final/supplementary figure S1-S4.pdf]

International Journal of
Molecular Sciences

MDPI

Supplementary Figure S1

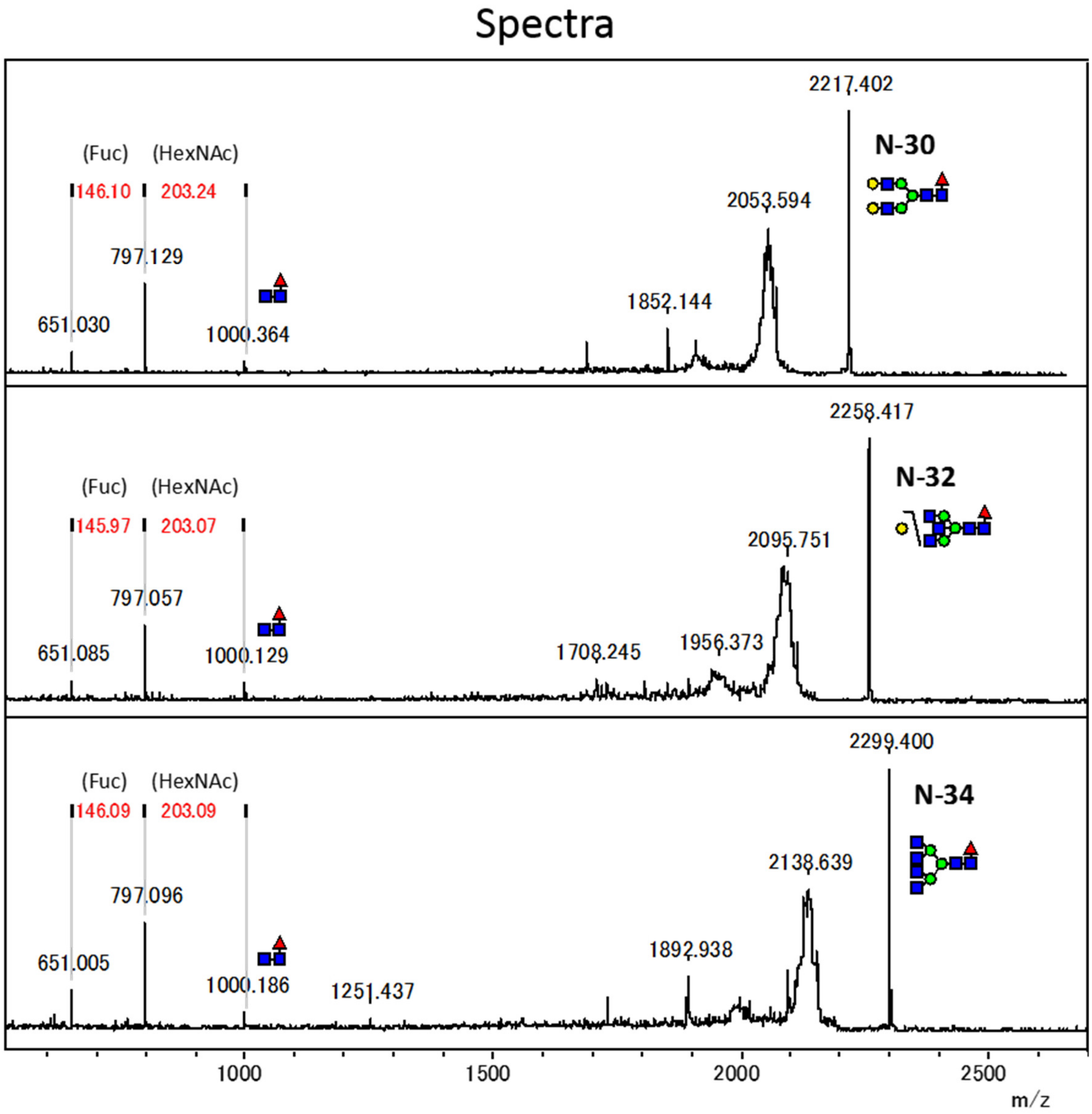

Figure S1. Highly expressed complex/hybrid neutral (C/H-N)-type glycans (N-30, -32, and -34) during chondrocyte hypertrophy. Most C/H-type glycans contain core fucose. (Day 0, 10, 20 from the Top).

Supplementary Figure S2

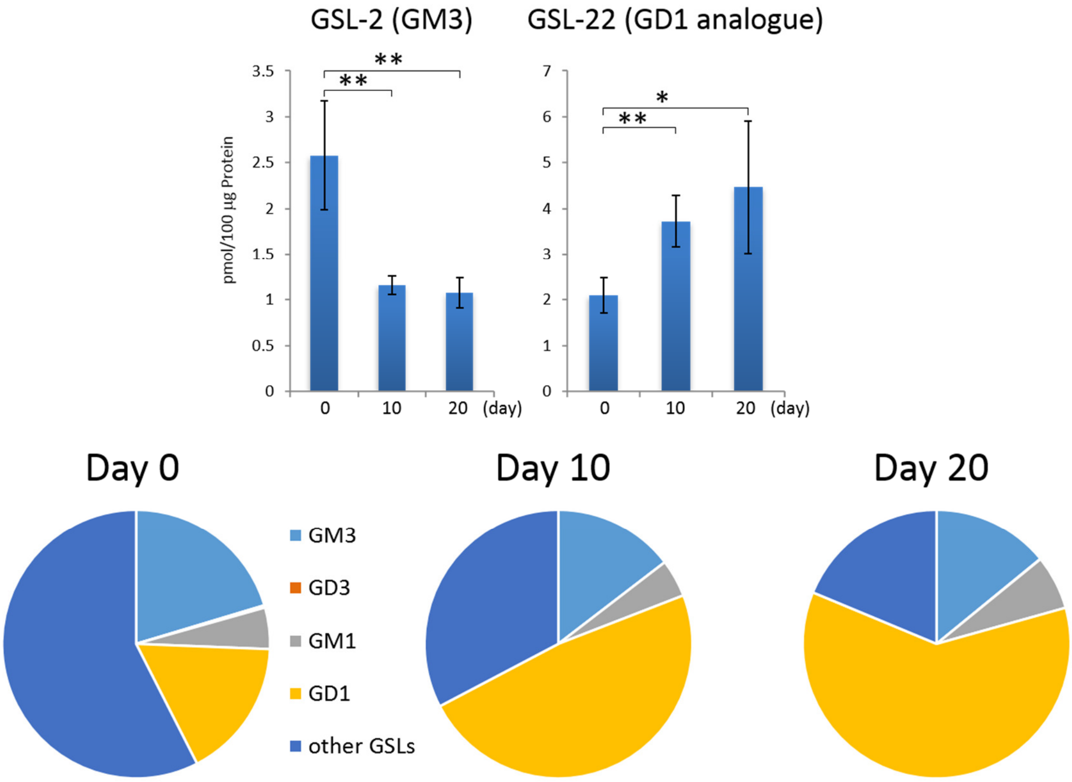

Figure S2. GM3 and GD1 analogues (Hex3HexNAc1Neu5Ac2) were the most abundant gangliosides in chondrocyte isolated from immature mice (lower). Increased GD1 analogues, associated with decreased expression of GM3 were shown in upper. The results are expressed as the means ± standard deviation (S.D.). *, $0.01 < p < 0.05$; **, $p < 0.01$. All MALDI-TOF-MS measurements were independently carried out ($n = 3$).

Supplementary Figure S3

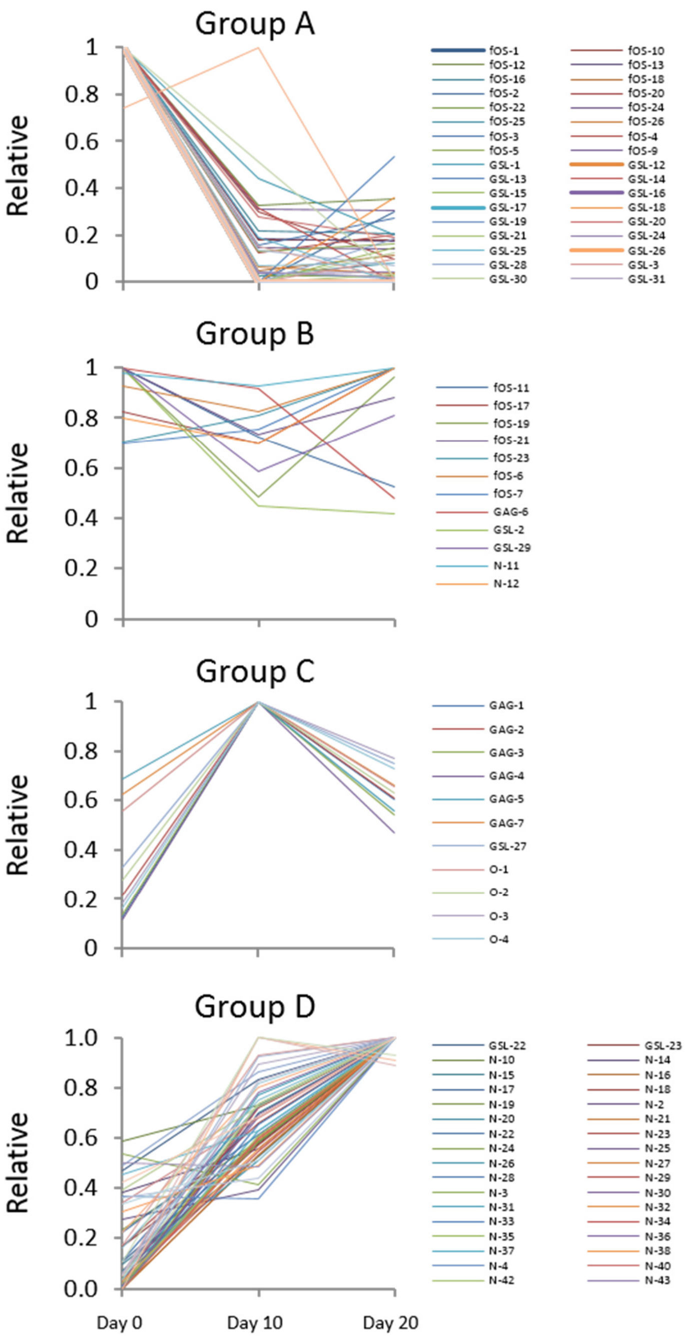

Figure S3. The change in the relative abundance of each glycan (classified into Group A, B, C, or D). The information of grouping by cluster analysis is listed in Table S1 associate it with glycan structures.

Supplementary Figure S4

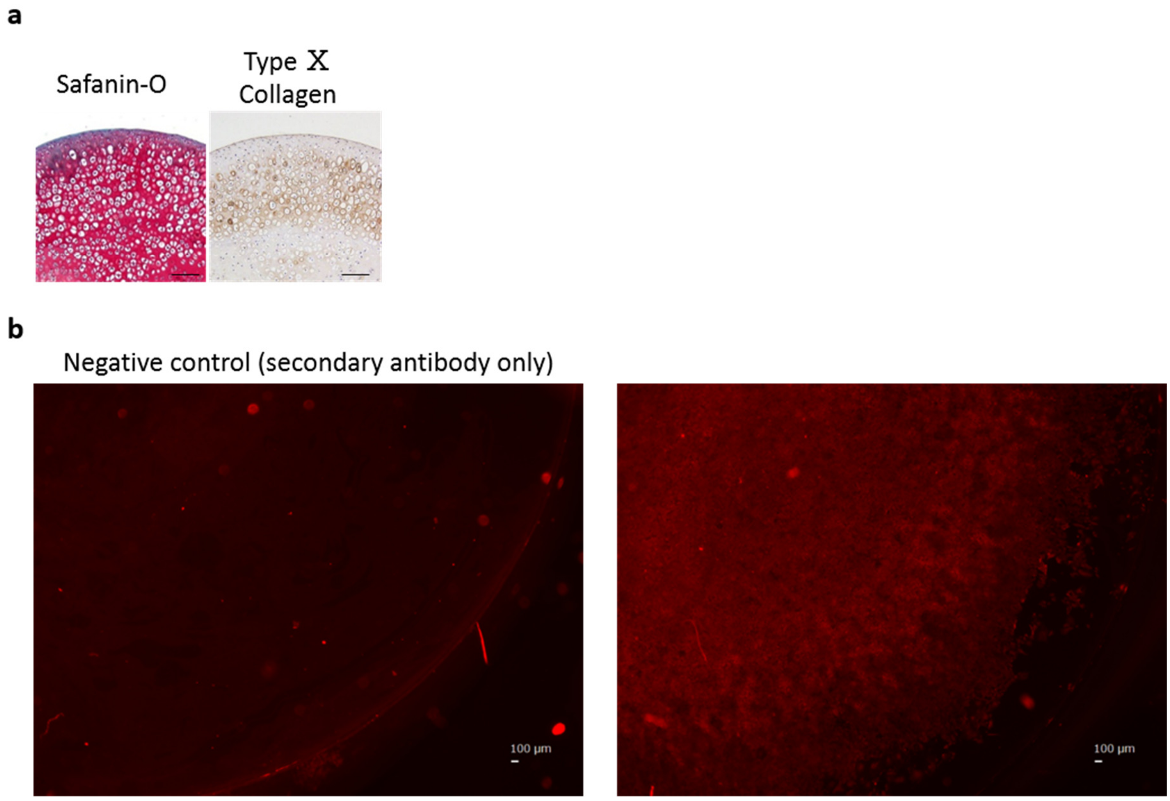

Figure S4. a. Mouse articular cartilage tissue was stained by the antibody against type X collagen as positive control. **b.** Negative control were also confirmed using only secondary antibody. Scale bars 100 µm.
