# Supplementary material for: Alteration of the Total Cellular Glycome during Late Differentiation of Chondrocytes
Source: Int J Mol Sci. 2019 Jul 19;20(14):3546. doi: 10.3390/ijms20143546 (PMC6678350; doi:10.3390/ijms20143546)
Supplement: Supplementary file 1 [file ijms-20-03546-s001.zip › ijms-532605-supplementary-final/supplementary table S1.pdf]

Table S1. Structure and amount of glycoconjugates in the total cellular glycomeof chondrocyte during hypertrophic differentiation.

No	Composition	Cluster Group	pmol/100 μg Protein Day0	pmol/100 μg Protein Day10	pmol/100 μg Protein Day20	*P*-value Day10/Day0	*P*-value Day20/Day0	Colors of Pie charts
N-1	(Hex)2 (HexNAc)2	A	0.175 ± 0.153	0.000 ± 0.000	0.000 ± 0.000	1.E-01	1.E-01	
N-2	(Hex)2 (HexNAc)2 (Fuc)1	D	1.361 ± 0.149	1.932 ± 0.464	4.902 ± 1.119	1.E-01	6.E-03	
N-3	(Hex)3 (HexNAc)2	D	2.293 ± 0.291	1.775 ± 0.497	4.290 ± 0.906	2.E-01	2.E-02	
N-4	(Hex)3 (HexNAc)2 (Fuc)1	D	4.695 ± 0.224	4.580 ± 1.426	12.844 ± 2.503	9.E-01	5.E-03	
N-5	(Hex)4 (HexNAc)2	D	2.221 ± 0.160	2.142 ± 0.287	4.421 ± 0.803	7.E-01	1.E-02	
N-6	(Hex)4 (HexNAc)2 (Fuc)1	D	0.506 ± 0.138	0.616 ± 0.147	1.397 ± 0.123	4.E-01	1.E-03	
N-7	(Hex)2 (Fuc)1 + (Man)3(GlcNAc)2	-	0.234 ± 0.203	0.307 ± 0.268	1.161 ± 0.274	7.E-01	9.E-03	
N-8	(Hex)2 + (Man)3(GlcNAc)2	D	15.658 ± 0.923	23.833 ± 4.665	46.683 ± 11.695	4.E-02	1.E-02	
N-9	(Hex)3 + (Man)3(GlcNAc)2	D	36.507 ± 1.500	58.781 ± 6.604	86.266 ± 18.948	5.E-03	1.E-02	
N-10	(Hex)4 + (Man)3(GlcNAc)2	D	27.452 ± 0.754	34.093 ± 3.773	46.788 ± 8.121	4.E-02	1.E-02	
N-11	(Hex)5 + (Man)3(GlcNAc)2	B	72.811 ± 3.410	68.821 ± 9.875	74.251 ± 13.931	5.E-01	9.E-01	
N-12	(Hex)6 + (Man)3(GlcNAc)2	B	57.040 ± 5.054	41.776 ± 9.049	60.161 ± 10.612	6.E-02	7.E-01	
N-13	(HexNAc)1 + (Man)3(GlcNAc)2	-	0.251 ± 0.217	0.366 ± 0.318	1.430 ± 0.401	6.E-01	1.E-02	
N-14	(HexNAc)1 (Fuc)1 + (Man)3(GlcNAc)2	D	1.180 ± 0.139	1.717 ± 0.215	3.073 ± 0.485	2.E-02	3.E-03	
N-15	(Hex)1 (HexNAc)1 + (Man)3(GlcNAc)2	D	0.199 ± 0.186	1.078 ± 0.114	2.057 ± 0.288	2.E-03	7.E-04	
N-16	(HexNAc)2 + (Man)3(GlcNAc)2	D	0.000 ± 0.000	0.962 ± 0.071	1.806 ± 0.252	2.E-05	2.E-04	
N-17	(Hex)1 (HexNAc)1 (Fuc)1 + (Man)3(GlcNAc)2	D	0.429 ± 0.373	2.277 ± 0.316	3.906 ± 0.638	3.E-03	1.E-03	
N-18	(Hex)2 (HexNAc)1 + (Man)3(GlcNAc)2	D	0.318 ± 0.276	2.543 ± 0.336	4.434 ± 0.199	9.E-04	3.E-05	
N-19	(HexNAc)2 (Fuc)1 + (Man)3(GlcNAc)2	D	2.642 ± 0.168	6.551 ± 0.810	11.155 ± 2.442	1.E-03	4.E-03	
N-20	(Hex)1 (HexNAc)2 + (Man)3(GlcNAc)2	D	0.000 ± 0.000	4.536 ± 0.680	6.496 ± 0.782	3.E-04	1.E-04	
N-21	(HexNAc)3 + (Man)3(GlcNAc)2	D	0.000 ± 0.000	0.976 ± 0.195	1.843 ± 0.145	1.E-03	3.E-05	
N-22	(Hex)2 (HexNAc)1 (Fuc)1 + (Man)3(GlcNAc)2	D	0.869 ± 0.164	2.373 ± 0.544	3.864 ± 0.567	1.E-02	9.E-04	
N-23	(Hex)3 (HexNAc)1 + (Man)3(GlcNAc)2	D	0.628 ± 0.286	2.476 ± 0.557	3.774 ± 0.410	7.E-03	4.E-04	
N-24	(Hex)1 (HexNAc)2 (Fuc)1 + (Man)3(GlcNAc)2	D	1.331 ± 0.203	19.465 ± 2.615	33.386 ± 7.469	3.E-04	2.E-03	
N-25	(Hex)2 (HexNAc)2 + (Man)3(GlcNAc)2	D	0.681 ± 0.065	8.210 ± 1.234	11.840 ± 1.728	5.E-04	4.E-04	
N-26	(HexNAc)3 (Fuc)1 + (Man)3(GlcNAc)2	D	0.781 ± 0.245	5.545 ± 1.026	10.894 ± 2.110	1.E-03	1.E-03	
N-27	(Hex)1 (HexNAc)3 + (Man)3(GlcNAc)2	D	0.000 ± 0.000	2.002 ± 0.444	3.314 ± 0.178	1.E-03	6.E-06	
N-28	(Hex)3 (HexNAc)1 (Fuc)1 + (Man)3(GlcNAc)2	D	0.166 ± 0.147	1.061 ± 0.268	1.452 ± 0.071	7.E-03	2.E-04	
N-29	(Hex)1 (HexNAc)2 (Fuc)2 + (Man)3(GlcNAc)2	D	0.000 ± 0.000	1.043 ± 0.326	1.454 ± 0.246	5.E-03	5.E-04	
N-30	(Hex)2 (HexNAc)2 (Fuc)1 + (Man)3(GlcNAc)2	D	2.046 ± 0.287	44.622 ± 6.195	61.611 ± 13.679	3.E-04	2.E-03	
N-31	(Hex)3 (HexNAc)2 + (Man)3(GlcNAc)2	D	0.000 ± 0.000	2.150 ± 0.462	2.791 ± 0.350	1.E-03	2.E-04	
N-32	(Hex)1 (HexNAc)3 (Fuc)1 + (Man)3(GlcNAc)2	D	0.804 ± 0.139	23.880 ± 3.917	40.316 ± 8.875	5.E-04	2.E-03	
N-33	(Hex)2(HexNAc)3+(Man)3(GlcNAc)2	D	0.039 ± 0.068	2.364 ± 0.688	3.627 ± 0.384	4.E-03	9.E-05	
N-34	(HexNAc)4 (Fuc)1 + (Man)3(GlcNAc)2	D	0.735 ± 0.149	21.567 ± 3.438	39.491 ± 8.895	5.E-04	2.E-03	
N-35	(Hex)2 (HexNAc)2 (Fuc)2 + (Man)3(GlcNAc)2	D	0.000 ± 0.000	0.881 ± 0.237	1.195 ± 0.150	3.E-03	2.E-04	
N-36	(Hex)3 (HexNAc)2 (Fuc)1 + (Man)3(GlcNAc)2	D	0.213 ± 0.189	9.942 ± 1.765	14.499 ± 3.113	7.E-04	1.E-03	
N-37	(Hex)4 (HexNAc)2 + (Man)3(GlcNAc)2	D	0.000 ± 0.000	1.261 ± 0.328	2.016 ± 0.241	3.E-03	1.E-04	
N-38	(Hex)2 (HexNAc)3 (Fuc)1 + (Man)3(GlcNAc)2	D	0.486 ± 0.144	9.501 ± 1.639	17.026 ± 3.467	7.E-04	1.E-03	
N-39	(Hex)3 (HexNAc)3 + (Man)3(GlcNAc)2	-	0.186 ± 0.170	0.559 ± 0.484	0.000 ± 0.000	3.E-01	1.E-01	
N-40	(HexNAc)4 (Fuc)2 + (Man)3(GlcNAc)2	D	0.000 ± 0.000	2.002 ± 0.498	3.363 ± 0.651	2.E-03	9.E-04	
N-41	(Hex)1(HexNAc)4 (Fuc)1 + (Man)3(GlcNAc)2	-	0.000 ± 0.000	0.771 ± 0.680	0.889 ± 0.779	1.E-01	1.E-01	
N-42	(Hex)4 (HexNAc)2 (Fuc)1 + (Man)3(GlcNAc)2	D	0.148 ± 0.134	4.410 ± 0.748	7.270 ± 1.451	6.E-04	1.E-03	
N-43	(Hex)3 (HexNAc)3 (Fuc)1 + (Man)3(GlcNAc)2	D	0.101 ± 0.088	1.534 ± 0.324	1.964 ± 0.267	2.E-03	3.E-04	
N-44	(Hex)3 (HexNAc)4 + (Man)3(GlcNAc)2	A	0.071 ± 0.067	0.000 ± 0.000	0.000 ± 0.000	1.E-01	1.E-01	
N-45	(Hex)1 (HexNAc)1 (Neu5Ac)1 + (Man)3(GlcNAc)2	D	0.743 ± 0.217	1.031 ± 0.358	1.641 ± 0.085	3.E-01	3.E-03	
N-46	(Hex)1 (HexNAc)1 (Fuc)1 (Neu5Ac)1 + (Man)3(GlcNAc)2	D	0.975 ± 0.115	1.565 ± 0.398	3.180 ± 0.612	7.E-02	4.E-03	
N-47	(Hex)2 (HexNAc)1 (Neu5Ac)1 + (Man)3(GlcNAc)2	D	2.004 ± 0.511	3.530 ± 0.747	4.097 ± 0.272	4.E-02	3.E-03	
N-48	(Hex)2 (HexNAc)1 (Fuc)1 (Neu5Ac)1 + (Man)3(GlcNAc)2	D	0.862 ± 0.150	1.724 ± 0.480	2.533 ± 0.338	4.E-02	1.E-03	
N-49	(Hex)3 (HexNAc)1 (Neu5Ac)1 + (Man)3(GlcNAc)2	D	1.047 ± 0.371	1.940 ± 0.673	2.667 ± 0.155	1.E-01	2.E-03	
N-50	(Hex)1 (HexNAc)2 (Fuc)1 (Neu5Ac)1 + (Man)3(GlcNAc)2	D	0.723 ± 0.148	3.321 ± 0.688	4.413 ± 0.735	3.E-03	1.E-03	
N-51	(Hex)2 (HexNAc)2 (Neu5Ac)1 + (Man)3(GlcNAc)2	D	0.911 ± 0.069	3.258 ± 0.713	4.075 ± 0.492	5.E-03	4.E-04	
N-52	(Hex)1 (HexNAc)2 (Fuc)2 (Neu5Ac)1 + (Man)3(GlcNAc)2	-	0.093 ± 0.087	0.000 ± 0.000	0.540 ± 0.472	1.E-01	2.E-01	
N-53	(Hex)2 (HexNAc)2 (Fuc)1 (Neu5Ac)1 + (Man)3(GlcNAc)2	D	1.876 ± 0.388	32.466 ± 5.285	35.163 ± 6.644	6.E-04	1.E-03	
N-54	(Hex)3 (HexNAc)2 (Neu5Ac)1 + (Man)3(GlcNAc)2	D	0.598 ± 0.188	3.157 ± 0.886	3.405 ± 0.442	8.E-03	5.E-04	
N-55	(Hex)1 (HexNAc)3 (Fuc)1 (Neu5Ac)1 + (Man)3(GlcNAc)2	D	0.517 ± 0.127	7.188 ± 1.498	9.808 ± 2.095	2.E-03	2.E-03	
N-56	(Hex)2 (HexNAc)2 (Neu5Ac)2 + (Man)3(GlcNAc)2	A	0.653 ± 0.113	0.000 ± 0.000	0.000 ± 0.000	6.E-04	6.E-04	
N-57	(Hex)3 (HexNAc)2 (Fuc)1 (Neu5Ac)1 + (Man)3(GlcNAc)2	D	0.822 ± 0.211	11.741 ± 2.105	13.113 ± 2.832	9.E-04	2.E-03	
N-58	(Hex)2 (HexNAc)3 (Fuc)1 (Neu5Ac)1 + (Man)3(GlcNAc)2	D	0.139 ± 0.122	3.093 ± 0.534	3.785 ± 0.581	7.E-04	4.E-04	
N-59	(Hex)2 (HexNAc)2 (Fuc)1 (Neu5Ac)2 + (Man)3(GlcNAc)2 lacton	D	0.000 ± 0.000	2.071 ± 0.509	1.879 ± 0.215	2.E-03	1.E-04	
N-60	(Hex)2 (HexNAc)2 (Fuc)1 (Neu5Ac)2 + (Man)3(GlcNAc)2	D	0.534 ± 0.132	10.442 ± 2.311	9.256 ± 2.159	2.E-03	2.E-03	
N-61	(Hex)3 (HexNAc)3 (Fuc)1 (Neu5Ac)1 + (Man)3(GlcNAc)2	D	0.153 ± 0.136	1.448 ± 0.324	1.343 ± 0.164	3.E-03	6.E-04	
N-62	(Hex)2 (HexNAc)3 (Fuc)1 (Neu5Ac)2 + (Man)3(GlcNAc)2	D	0.058 ± 0.051	0.856 ± 0.204	1.038 ± 0.170	3.E-03	7.E-04	
N-63	(Hex)3 (HexNAc)3 (Neu5Ac)2 + (Man)3(GlcNAc)2	A	0.060 ± 0.052	0.000 ± 0.000	0.000 ± 0.000	1.E-01	1.E-01	
N-64	(Hex)3 (HexNAc)3 (Fuc)1 (Neu5Ac)2 + (Man)3(GlcNAc)2	-	0.087 ± 0.079	0.878 ± 0.268	0.421 ± 0.365	8.E-03	2.E-01	
N-65	(Hex)3 (HexNAc)3 (Fuc)1 (Neu5Ac)3 + (Man)3(GlcNAc)2	-	0.069 ± 0.064	0.429 ± 0.146	0.113 ± 0.195	2.E-02	7.E-01	
O-1	(GalNAc)1 (Tn)	C	102.283 ± 10.086	182.883 ± 46.954	119.933 ± 33.976	4.E-02	4.E-01	
O-2	(Gal)1(GalNAc)1 (T)	C	8.554 ± 0.294	30.797 ± 8.843	19.376 ± 0.300	1.E-02	2.E-06	
O-3	(Gal)1(GalNAc)1(Neu5Ac)1 (ST)	C	11.663 ± 1.251	64.104 ± 18.924	49.213 ± 3.474	9.E-03	6.E-05	
O-4	(Gal)1(GalNAc)1(Neu5Ac)2 (DST)	C	3.968 ± 0.128	24.018 ± 11.035	17.520 ± 3.582	3.E-02	3.E-03	
GSL-1	(Hex)2	A	11.887 ± 11.878	5.253 ± 0.366	2.384 ± 1.141	4.E-01	2.E-01	
GSL-2	(Hex)2(Neu5Ac)1	B	10.315 ± 2.383	4.637 ± 0.414	4.319 ± 0.659	2.E-02	1.E-02	
GSL-3	(Hex)2(Neu5Ac)2	A	0.142 ± 0.019	0.000 ± 0.000	0.014 ± 0.024	2.E-04	2.E-03	
GSL-4	(Hex)3(HexNAc)2(Neu5Ac)2	A	3.145 ± 0.566	0.226 ± 0.010	0.258 ± 0.096	9.E-04	1.E-03	
GSL-5	(Hex)3	A	0.140 ± 0.168	0.188 ± 0.326	0.002 ± 0.003	8.E-01	2.E-01	
GSL-6	(Hex)4(HexNAc)1	A	2.034 ± 2.204	0.272 ± 0.089	0.143 ± 0.145	2.E-01	2.E-01	
GSL-7	(Hex)5(HexNAc)1	A	1.793 ± 3.105	0.275 ± 0.163	0.026 ± 0.046	4.E-01	4.E-01	
GSL-8	(Hex)7	-	0.000 ± 0.000	0.000 ± 0.000	0.001 ± 0.002	n.d.	4.E-01	
GSL-9	(Hex)6(HexNAc)1	A	0.074 ± 0.128	0.038 ± 0.044	0.002 ± 0.003	7.E-01	4.E-01	
GSL-10	(Hex)7(HexNAc)1	-	0.001 ± 0.002	0.029 ± 0.026	0.000 ± 0.000	1.E-01	4.E-01	
GSL-11	(Hex)8(HexNAc)1	-	0.000 ± 0.000	0.012 ± 0.020	0.000 ± 0.000	4.E-01	n.d.	
GSL-12	(Hex)5(HexNAc)3	A	0.040 ± 0.045	0.000 ± 0.000	0.000 ± 0.000	2.E-01	2.E-01	
GSL-13	(Hex)4(HexNAc)4	A	0.025 ± 0.043	0.000 ± 0.000	0.013 ± 0.023	4.E-01	7.E-01	
GSL-14	(Hex)4(HexNAc)3(Neu5Ac)1	A	0.144 ± 0.170	0.046 ± 0.039	0.000 ± 0.000	4.E-01	2.E-01	
GSL-15	(Hex)5(HexNAc)3(Neu5Ac)1	A	0.042 ± 0.023	0.000 ± 0.000	0.006 ± 0.011	3.E-02	7.E-02	
GSL-16	(Hex)6(HexNAc)3(Neu5Ac)1	A	0.034 ± 0.029	0.000 ± 0.000	0.000 ± 0.000	1.E-01	1.E-01	
GSL-17	(Hex)5(HexNAc)4(Neu5Ac)1	A	0.028 ± 0.048	0.000 ± 0.000	0.000 ± 0.000	4.E-01	4.E-01	
GSL-18	(Hex)6(HexNAc)3(Fuc)4	A	0.034 ± 0.031	0.000 ± 0.000	0.012 ± 0.021	1.E-01	4.E-01	
GSL-19	(Hex)5(HexNAc)2	A	0.611 ± 0.206	0.024 ± 0.030	0.010 ± 0.012	8.E-03	7.E-03	
GSL-20	(Hex)3(HexNAc)1	A	7.091 ± 1.840	1.979 ± 0.109	1.348 ± 0.432	9.E-03	6.E-03	
GSL-21	(Hex)3(HexNAc)2(Neu5Ac)1	A	0.471 ± 0.107	0.000 ± 0.000	0.015 ± 0.014	2.E-03	2.E-03	
GSL-22	(Hex)3(HexNAc)1(Neu5Ac)2	D	8.417 ± 1.537	14.888 ± 2.256	17.846 ± 5.795	1.E-02	5.E-02	
GSL-23	(Hex)3(HexNAc)1(Neu5Ac)1(Neu5Gc)1	D	0.115 ± 0.013	0.398 ± 0.054	0.691 ± 0.282	9.E-04	2.E-02	
GSL-24	(Hex)4(HexNAc)2(Neu5Ac)1	A	0.220 ± 0.018	0.008 ± 0.014	0.009 ± 0.016	8.E-05	1.E-04	
GSL-25	(Hex)3(HexNAc)1(Neu5Ac)3-lactone2	A	0.078 ± 0.034	0.015 ± 0.026	0.000 ± 0.000	6.E-02	2.E-02	
GSL-26	(Hex)3(HexNAc)1(Neu5Ac)3-lactone1	A	0.240 ± 0.030	0.000 ± 0.000	0.000 ± 0.000	2.E-04	2.E-04	
GSL-27	(Hex)3(HexNAc)2	C	0.654 ± 0.686	1.991 ± 0.293	1.490 ± 0.415	4.E-02	1.E-01	
GSL-28	(Hex)3(HexNAc)1(Neu5Ac)1-lactone1	A	0.079 ± 0.019	0.000 ± 0.000	0.014 ± 0.025	2.E-03	2.E-02	
GSL-29	(Hex)3(HexNAc)1(Neu5Ac)1	B	2.442 ± 0.534	1.434 ± 0.294	1.982 ± 0.730	5.E-02	4.E-01	
GSL-30	(Hex)4(HexNAc)1(Neu5Ac)1	A	0.118 ± 0.015	0.000 ± 0.000	0.015 ± 0.025	2.E-04	4.E-03	
GSL-31	(Hex)3(HexNAc)3(Fuc)1	A	0.221 ± 0.365	0.010 ± 0.009	0.005 ± 0.009	4.E-01	4.E-01	
fOS-1	(Hex)1 (HexNAc)1	A	1.345 ± 0.254	0.000 ± 0.000	0.000 ± 0.000	8.E-04	8.E-04	
fOS-2	(Hex)2 (HexNAc)1	A	2.482 ± 0.402	0.000 ± 0.000	0.744 ± 0.126	4.E-04	2.E-03	
fOS-3	(Hex)3 (HexNAc)1	A	3.667 ± 0.648	0.580 ± 0.264	0.990 ± 0.416	2.E-03	4.E-03	
fOS-4	(Hex)4 (HexNAc)1	A	12.608 ± 1.385	1.590 ± 0.712	2.531 ± 1.072	3.E-04	6.E-04	
fOS-5	(Hex)5 (HexNAc)1	A	19.144 ± 1.160	2.439 ± 0.359	3.147 ± 0.477	2.E-05	2.E-05	
fOS-6	(Hex)6 (HexNAc)1	B	0.911 ± 0.079	0.811 ± 0.288	0.984 ± 0.150	6.E-01	5.E-01	
fOS-7	(Hex)7 (HexNAc)1	B	0.241 ± 0.035	0.261 ± 0.180	0.347 ± 0.121	9.E-01	2.E-01	
fOS-8	(Hex)8 (HexNAc)1	-	0.000 ± 0.000	0.133 ± 0.119	0.191 ± 0.022	1.E-01	1.E-04	
fOS-9	(Hex)2 (HexNAc)2	A	1.416 ± 0.254	0.441 ± 0.033	0.434 ± 0.066	3.E-03	3.E-03	
fOS-10	(Hex)3 (HexNAc)2	A	2.239 ± 0.350	0.395 ± 0.016	0.399 ± 0.040	8.E-04	8.E-04	
fOS-11	(Hex)4 (HexNAc)2	B	1.166 ± 0.184	0.842 ± 0.028	0.613 ± 0.122	4.E-02	1.E-02	
fOS-12	(Hex)5 (HexNAc)2	A	0.514 ± 0.107	0.168 ± 0.047	0.180 ± 0.064	7.E-03	1.E-02	
fOS-13	(Hex)6 (HexNAc)2	A	0.210 ± 0.037	0.038 ± 0.066	0.036 ± 0.063	2.E-02	1.E-02	
fOS-14	(Hex)4 (HexNAc)1(Fuc)1	-	0.000 ± 0.000	0.021 ± 0.037	0.105 ± 0.022	4.E-01	1.E-03	
fOS-15	(HexNAc)1 (Fuc)1 + (Man)3(GlcNAc)1/(Hex)3 (HexNAc)2 (Fuc)1	-	0.068 ± 0.062	0.020 ± 0.035	0.161 ± 0.017	3.E-01	6.E-02	
fOS-16	(HexNAc)2 + (Man)3(GlcNAc)1/(HexNAc)1 + (Man)3(GlcNAc)2	A	1.084 ± 0.169	0.235 ± 0.022	0.225 ± 0.072	2.E-03	2.E-03	
fOS-17	(HexNAc)1(HexNAc)1+ (Man)3(GlcNAc)1	B	0.216 ± 0.005	0.184 ± 0.022	0.263 ± 0.031	7.E-02	6.E-02	
fOS-18	(HexNAc)2 (Fuc)1 + (Man)3(GlcNAc)1/(HexNAc)1 (Fuc)1 + (Man)3(GlcNAc)2	A	1.438 ± 0.270	0.065 ± 0.057	0.164 ± 0.040	1.E-03	1.E-03	
fOS-19	(Hex)1 (HexNAc)2 + (Man)3(GlcNAc)1/(Hex)1 (HexNAc)1 + (Man)3(GlcNAc)2	B	0.094 ± 0.081	0.045 ± 0.078	0.090 ± 0.078	5.E-01	1.E+00	
fOS-20	(HexNAc)3 + (Man)3(GlcNAc)1/(HexNAc)2 + (Man)3(GlcNAc)2	A	0.347 ± 0.069	0.104 ± 0.009	0.034 ± 0.059	4.E-03	4.E-03	
fOS-21	(Hex)1 (HexNAc)1 (Neu5Ac)1 + (Man)3(GlcNAc)1	B	0.170 ± 0.007	0.124 ± 0.017	0.149 ± 0.027	1.E-02	3.E-01	
fOS-22	(HexNAc)2 (Neu5Ac)1 + (Man)3(GlcNAc)1	A	22.345 ± 4.580	0.603 ± 0.032	0.505 ± 0.140	1.E-03	1.E-03	
fOS-23	(Hex)2 (HexNAc)2 + (Man)3(GlcNAc)1/(Hex)2 (HexNAc)1 + (Man)3(GlcNAc)2	B	0.073 ± 0.064	0.085 ± 0.074	0.105 ± 0.091	8.E-01	7.E-01	
fOS-24	(HexNAc)4 + (Man)3(GlcNAc)1/(HexNAc)3 + (Man)3(GlcNAc)2	A	0.170 ± 0.023	0.024 ± 0.042	0.024 ± 0.041	6.E-03	6.E-03	
fOS-25	(Hex)1 (HexNAc)2 (Neu5Ac)1 + (Man)3(GlcNAc)1/(Hex)1 (HexNAc)1 (Neu5Ac)1 + (Man)3(GlcNAc)2	A	1.406 ± 0.404	0.032 ± 0.056	0.110 ± 0.009	4.E-03	5.E-03	
fOS-26	(Hex)1 (HexNAc)2 (Neu5Ac)1 + (Man)3(GlcNAc)2	A	1.103 ± 0.352	0.068 ± 0.060	0.042 ± 0.073	7.E-03	7.E-03	
GAG-1	CS-0S	C	288.450 ± 54.372	2278.017 ± 36.359	1381.278 ± 255.549	7.77E-07	1.93E-03	
GAG-2	HA	C	82.687 ± 14.097	379.795 ± 14.307	230.571 ± 68.393	1.38E-05	2.14E-02	
GAG-3	CS-2S	C	17.012 ± 2.462	123.291 ± 2.347	66.964 ± 14.082	6.98E-07	3.76E-03	
GAG-4	CS-4S	C	1296.509 ± 209.632	10988.177 ± 1858.768	5162.548 ± 1113.825	8.53E-04	4.11E-03	
GAG-5	CS-6S	C	87.085 ± 15.004	126.781 ± 35.093	70.447 ± 11.446	1.46E-01	2.01E-01	
GAG-6	HS-2S	B	139.249 ± 96.725	128.011 ± 9.942	66.498 ± 31.982	8.51E-01	2.84E-01	
GAG-7	HS-2SNS	C	6.499 ± 1.527	10.414 ± 0.908	6.863 ± 2.871	1.88E-02	8.56E-01	
