# Supplementary material for: Alteration of the Total Cellular Glycome during Late Differentiation of Chondrocytes
Source: Int J Mol Sci. 2019 Jul 19;20(14):3546. doi: 10.3390/ijms20143546 (PMC6678350; doi:10.3390/ijms20143546)
Supplement: Supplementary file 1 [file ijms-20-03546-s001.zip › ijms-532605-supplementary-final/supplementary table S2.pdf]

Table S2. Primers used in this study.

Gene		Sequence
Runx2	Forward	GATGGGACTGTGGTTACCG
	Reverse	GGTGAAACTCTTGCCTCGTC
Sox9	Forward	CCCCGATTACAAGTACCAGC
	Reverse	AGCGCCTTGAAGATAGCATT
Mmp13	Forward	TTGGCCACTCCCTAGGTCTG
	Reverse	GGTTGGGGTCTTCATCGC
Col10a1	Forward	GGCTTCAGGGAGTGCAATC
	Reverse	CTCACATGGGAGCCACTAGG
Col2a1	Forward	AGGATGGCTGCACCAAACAC
	Reverse	TGTCCATGGGTGCGATGTC
Ihh	Forward	CTCTTGCCTACAAGCAGTTCA
	Reverse	CCGTGTTCTCCTCGTCCTT
Aggrecan	Forward	CCCTCACCCCAAGAATCAAG
	Reverse	GGATAGTTGGGGAGCGACAC
Adamts5	Forward	GCATGCGCGCTACACTCTAA
	Reverse	TCGCGGTTGTAGACATGCAG
Alp	Forward	GACTGGTACTCGGATAACGAGATGC
	Reverse	TGCGGTTCCAGACATAGTGG
Igf-1	Forward	AGGCTATGGCTCCAGCATTC
	Reverse	CTTCAGTGGGGCACAGTACA
Mgat1	Forward	AGGCCTATGACCGGGACTTC
	Reverse	GCTAGTGTACTGTACCCGCACCTC
Mgat2	Forward	ACCTACACCACCATTCGGAGTTTC
	Reverse	ATGCATCTCGGGTCAAGGCTA
Mgat3	Forward	ATGATTGCTCATTACCCTGCTCTC
	Reverse	ATTCCACAGGTAGCCCTGCATA
Fut8	Forward	AACAGCTTGTTAAGGCCAAAG
	Reverse	GCATGTCTTTGGAGTTCATTTC
Galnt3	Forward	ACACTATTTACCCGGAAGCG
	Reverse	AGCTCCTTCTGGATGTTGTG
Csgalnact1	Forward	CCAATTTCAGAAACTTCACCTTCAT
	Reverse	TGTTCAGCCTACAAGTGTTGAG
Csgalnact2	Forward	TTAATATCATTGTGCCACTTGCG
	Reverse	TAGAATAGACTTGACTTTAGATAGTCCTT
C4st-1	Forward	ACCTCGTGGGCAAGTATGAG
	Reverse	TCTGGAAGAACTCCGTGGTC
Gapdh	Forward	AGGTCGGTGTGAACGGATTTG
	Reverse	GGGGTCGTTGATGGCAACA
